# A new approach to characterize postural deficits in chemotherapy-induced peripheral neuropathy and to analyze postural adaptions after an exercise intervention

**DOI:** 10.1186/s12883-019-1589-7

**Published:** 2020-01-16

**Authors:** Sarah Kneis, Anja Wehrle, Daniela Dalin, Isabella Katharina Wiesmeier, Johann Lambeck, Albert Gollhofer, Hartmut Bertz, Christoph Maurer

**Affiliations:** 1 Department of Neurology and Neuroscience, Faculty of Medicine, Medical Center, University of Freiburg, Freiburg, Germany; 2 Institute for Exercise- and Occupational Medicine, Faculty of Medicine, Medical Center, University of Freiburg, Freiburg, Germany; 3 Department of Sports and Sport Science, University of Freiburg, Freiburg, Germany

**Keywords:** Postural stability, Chemotherapy-induced peripheral neuropathy, Motor control, Sensory weighting, Model

## Abstract

**Background:**

Postural instability presents a common and disabling consequence of chemotherapy-induced peripheral neuropathy (CIPN). However, knowledge about postural behavior of CIPN patients is sparse. With this pilot study, we used a new approach to i) characterize postural impairments as compared to healthy subjects, ii) allocate possible abnormalities to a set of parameters describing sensorimotor function, and iii) evaluate the effects of a balance-based exercise intervention.

**Methods:**

We analyzed spontaneous and externally perturbed postural control in eight CIPN patients before and after a balance-based exercise intervention by using a modification of an established postural control model. These findings were compared to 15 matched healthy subjects.

**Results:**

Spontaneous sway amplitude and velocity were larger in CIPN patients compared to healthy subjects. CIPN patients’ reactions to external perturbations were smaller compared to healthy subjects, indicating that patients favor vestibular over proprioceptive sensory information. The balance-based exercise intervention up-weighted proprioceptive information in patients.

**Conclusions:**

CIPN patients’ major postural deficit may relate to underuse of proprioceptive information that results in a less accurate posture control as spontaneous sway results indicate. The balance-based exercise intervention is able to partially correct for this abnormality. Our study contributes to a better understanding of postural impairments in CIPN patients and suggests an effective treatment strategy.

**Trial registration:**

German Clinical Trials Register: DRKS00004340, retrospectively registered 04 January 2013.

## Clinical message

- CIPN patients present greater postural sway than healthy control subjects associated with postural instability
- CIPN patients use less proprioceptive information than control subjects entailing less accuracy for posture control
- A balance-based exercise intervention can partially correct for the proprioceptive underuse of CIPN patients

## Background

Chemotherapy-induced peripheral neuropathy (CIPN) is a common and clinically relevant side-effect of cancer treatment [1–3]. CIPN can cause treatment delays and dose reductions, interfering with general outcome or compromising survival [3–6]. Consequences of CIPN can lead to excessive healthcare costs and resource use [7]. Symptoms of CIPN primarily include paraesthesia, dysesthesia, numbness and pain with a symmetric, distal, length-dependent “glove and stocking” distribution [3, 8] and limit patients’ everyday life considerably. Additionally, CIPN patients often suffer from postural instability [9–17], contributing to a lower quality of life [5, 18], a higher risk of mortality [19–22] and increased healthcare costs [23, 24].

Quantitative reports about CIPN patients’ postural instability are currently rising: CIPN has been associated with reduced gait abilities [13, 14] and changes in center of pressure (COP) displacements [10, 12, 25]. However, underlying mechanisms are sparsely described [10, 25]: Wampler et al. [10] assumed that besides somatosensory impairments also diminished vestibular function causes increased postural sway in CIPN patients. Furthermore, in an earlier study we found changes in elicitability and sensitivity of spinal reflex circuitry associated with postural instability in CIPN patients [25]. More comprehensive knowledge about neuropathy-induced postural instability has been derived from patients diagnosed with diabetes: Bonnet et al. [26] deduced larger COP displacements, which were more pronounced with visual disturbance. Diabetic neuropathy patients seem to delay postural reactions [27], shift from ankle to hip strategy [26, 28, 29], and seem to use vestibular rather than proprioceptive cues [30]. However, proprioception may be essential for stability in both quiet stance and during unexpected postural perturbations [26, 31–33], since it provides information about lower limb orientation with respect to the support base [34, 35]. They report a clear increase in postural sway when proprioceptive cues are deficient [10, 26, 32]. Our first aim here is to characterize the postural deficits in CIPN and to extract the sensorimotor abnormalities using a well-established model of postural control [36–38].

Concerning treatment, knowledge about the management of CIPN-induced postural instability is still sparse [39–41]. Generally, it is increasingly suggested to focus on strength and especially balance exercises in order to improve physical functioning of CIPN patients [11], what we could confirm in a randomized controlled trial by ourselves [42]. Until recently, there are only two other interventional studies showing that balance exercises improved CIPN-related postural control deficits [39, 41]. However, evidence from diabetes research on neuropathy further supports this assumption [43–45]. Balance training in general has proven to enhance postural stability by inducing neuronal adaptations and improving muscular output [46, 47]. Hence, we aimed to implement a balance-based exercise intervention for CIPN patients.

In sum, the present study was undertaken to i) specify the postural abnormalities associated with CIPN during spontaneous and externally perturbed stance, ii) to identify the underlying sensorimotor malfunction, and iii) to monitor the effect of a balance-based exercise intervention in a pilot approach.

## Methods

### Patients

The present pilot study provides two approaches: a cross-sectional approach to identify postural-control differences between CIPN patients and matched healthy control subjects and a one-armed longitudinal approach to evaluate the effects of a balance-based exercise intervention on CIPN-related postural deficits.

Therefore, we examined eight cancer patients with different cancer localizations and treatment status, all reporting severe neuropathy symptoms due to chemotherapy (CIPN). The chemotherapies applied entailed the neurotoxic agents bortezomib, carboplatin, cisplatin, paclitaxal, docetaxal and vincristine. None of the patients had any neuropathy symptom before the application of neurotoxic agents. CIPN was clinically and electrophysiologically confirmed in all patients. Moreover, we assessed patients’ subjective CIPN symptoms via the neurotoxicity subscale (NtxS) of FACT&GOG (Functional Assessment of Cancer Therapy/Gynaecology Oncology Group) scored from 0 to 44 (0 = severe symptoms; 44 = no symptoms); Table 1 summarizes our patients’ clinical information.

**Table 1:** Subjects’ characteristic

									Electrophysiology		Reflexes		Vibration sense		
Patient	Sex	Height (cm)	Weight (kg)	Age (years)	Diagnosis	Status	Months since initial diagnosis (n)	Cycles of neurotoxic drugs (n)	NCV N.tibialis (m/s)	APA N.suralis (μV)	Achilles tendon	Patellar tendon	right	left	NtxS (score)
1	m	182	125	55–59	B-NHL	Partial remission	185	14	32	1.7	–	–	6/8	6/8	34
2	m	182	80	65–69	Multiple myeloma	Stable disease	58	34	0	0	–	+	5.5/8	0.5/8	29
3	f	161	86	60–64	Multiple myeloma	Relapse	82	7	28	6.05	–	–	4/8	3.5/8	24
4	f	162	60	65–69	Breast cancer	Stable disease	66	19	49	3.55	–	+	5/8	5/8	23
5	m	180	80	60–64	Multiple myeloma	Stable disease	8	4	39	1.55	(−)	(−)	0.5/8	1.5/8	34
6	m	167	92	60–64	Breast cancer	Stable disease	195	25	40	0	–	–	0.5/8	4/8	14
7	m	176	64	65–69	Rectal cancer	Progressive disease	118	79	37	0	–	–	0/8	0/8	23
8	m	188	107	40–44	Germ cell cancer	Complete remission	66	4	42	1	–	–	4/8	0/8	22
mean ± SD	m:f 6:2	174.8 ± 10.1	86.8 ± 21.5	60.8 ± 8.7			97.3 ± 64.8	23.3 ± 24.9	34.4 ± 14.9	1.7 ± 2.1			3.2 ± 2.5	2.6 ± 2.4	25.4 ± 6.3
Healthy control subjects															
mean ± SD	m:f 9:6	17.0 ± 9.1	78.0 ± 6.3	59.6 ± 10.0											

*APA* action potential amplitude (normal values: ≥6 μV), *B-NHL* B-Non Hodgkin Lymphoma, *f* female, *m* male, *NCV* nerve conduction velocity (normal values: ≥41 m/s), *NtxS* neurotoxicity subscale of FACT/GOG (Functional Assessment of Cancer Therapy/Gynaecology Oncology Group) scored from 0 (severe symptoms) – 44 (no symptoms); SD, standard deviation; “-”, no reflex; “(−)”, reduced reflex; “+”, normal reflex

We excluded patients with other possible sources of neuropathy (eg hereditary, diabetes- or alcohol-induced) and patients suffering from additional deficits that might interact with their postural control such as a relevant reduction of muscular strength or certain comorbidities (eg osteolysis, severe vertebral degeneration, vestibular deficits). Specifically, all patients underwent detailed vestibular testing using a rotating chair. In addition, patients performed an incremental stress electrocardiogram on a stationary bicycle in the Institute for Exercise- and Occupational Medicine, Medical Center – University of Freiburg in order to exclude cardiovascular risks during exercise and to determine the lactate threshold for exercise control.

The control group for the postural control experiments consisted of 15 healthy subjects matched to patients’ age, weight and height. We assigned two matches to each patient (except for one patient with a relative heavy body weight) to ensure a more reliable representation of the postural behavior of healthy subjects.

Patients underwent assessments of posture control twice (before and after 12 weeks of a supervised exercise intervention) while healthy control subjects underwent the assessment only once.

Patients’ recruitment and data collection took place in the Clinic of Internal Medicine I and posture analyses and clinical assessments took place in the Department of Neurology and Clinical Neurophysiology, Medical Center – University of Freiburg.

The study was approved by the Ethics Commission of University of Freiburg. All subjects provided written informed consent to the experimental procedure in accordance with the Declaration of Helsinki.

### Intervention

The one-on-one training sessions took place in the division of Sports Oncology in the Clinic of Internal Medicine I, twice per week over 12 weeks. The intervention protocol included a cardiovascular warm-up of up to 20 min on a stationary bicycle with an intensity of 75–80% of maximum heart rate, followed by the balance-based exercises for 30 min and muscular endurance training for the main muscle groups. The main focus was on the balance part of the training. Balance training prescription included a progressive increase over the intervention period in the exercise amount and difficulty. Depending on the individual performance level, that could vary during the interventions period, patient performed three (beginners) to eight exercises (more advanced) with three repetitions each à 20–30s (a 20-s rest between the repetitions and a 2-min rest between the different exercises to avoid fatigue). Moreover, exercise difficulty was also adapted to patients’ performance level and successively increased by reducing the support surface (eg bipedal- to mono-pedal stance) and visual input (eyes closed), adding motor/cognitive tasks (eg moving arms or counting backwards) and inducing instability (throwing a ball or being perturbed by the sports therapist) to stimulate the sensorimotor system adequately [46, 48]. We documented vital parameters, training progress, and reasons for missed sessions.

### Procedure and data analysis

**Fig. 1:**
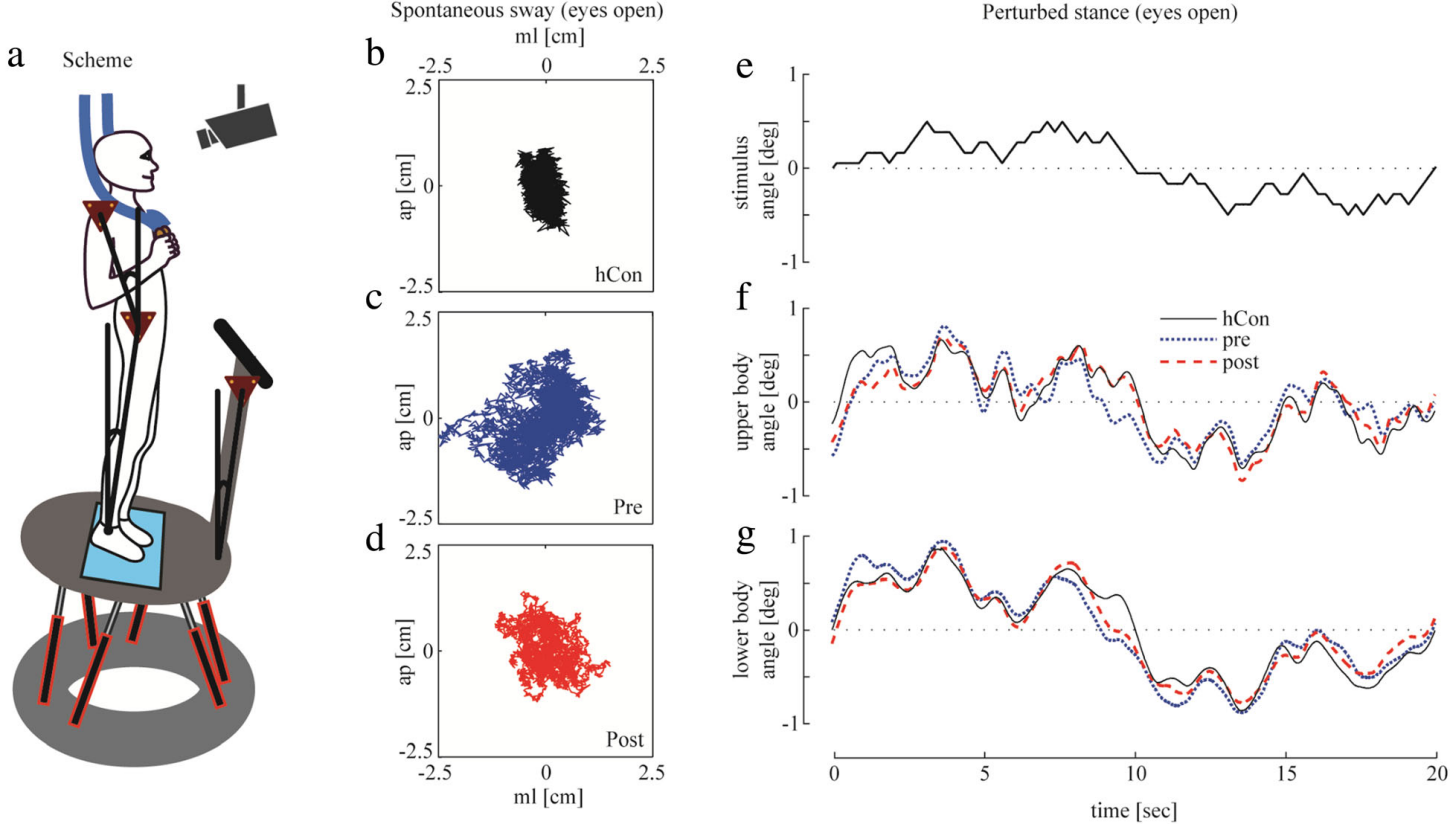
Experimental setup. *Scheme:* of a subject standing on the platform in an upright position **a**. *Spontaneous sway*: COP displacement of one representative subject of the control subjects’ group (**b**, hCon) and the patients’ group **c** before (pre) and **d** after (post) intervention in the eyes open condition. *Perturbed stance*: **e** (stimulus) 1° peak-to-peak platform rotation; postural reactions of **f** upper body and **g** lower body over 20 s with eyes open in a representative healthy control subject (hCon) and a patient before (pre) and after (post) intervention. Body reaction (**f**, **g**) follows the platform movement (**e**). deg, degrees, sec, seconds, ap, anterior-posterior, ml, medio-lateral, cm, centimeter

For evaluating postural control*, spontaneous sway* and *perturbed stance* were measured with a custom-built motion platform [49, 50] under two visual conditions, with eyes open and with eyes closed. Each trial lasted 1 minute. The participants were told to stand upright on the platform in comfortable shoes. Stance width was predetermined within a marked area. For safety reasons, participants had to hold two ropes hanging from the ceiling in a crossed-arms position so that they could not perceive a somatosensory spatial orientation signal (Fig. 1a).

Data analysis was conducted off-line with custom-made software programmed in MATLAB® (The MathWorks Inc., Natick, MA, USA).

*Spontaneous sway* was measured on the non-moving platform. The center of pressure (*COP*) sway path was detected with a force transducing platform (Fig. 1b-d, Kistler platform type 9286, Winterthur, Switzerland). From the COP excursions over time in anterior-posterior and medio-lateral *sway directions,* we calculated the root mean square (*RMS*) around the mean COP position. After differentiating the time series, we calculated mean velocity (*MV*). In addition, center frequency (*CF*) was extracted from the power spectrum [51, 52].

*Perturbed stance* was measured on the moving platform to differentiate sensory contributions in reaction to external disturbances. We analyzed rotational tilts in the sagittal plane with the tilt axis passing through the participant’s ankle joints. Platform rotations were designed as pseudorandom stimuli (PRTS, pseudorandom ternary sequence, see Fig. 1e) [53]. This stimulus has a wide spectral bandwidth with the velocity waveform having spectral and statistical properties approximating a white noise stimulus [53]. As such, this stimulus appeared to be unpredictable to the test subject. We applied two peak angular displacements (*stimulus amplitude*: 0.5° and 1° peak-to-peak) and analyzed at eleven *stimulus frequencies* (0.05, 0.15, 0.3, 0.4, 0.55, 0.7, 0.9, 1.1, 1.35, 1.75 and 2.2 Hz).

Angular excursions of the lower (hip-to-ankle: hip movement) and upper (shoulder-to-hip: shoulder movement) *body segments* and the platform in space were measured using an optoelectronic motion-measuring device with markers attached to shoulder and hip (Optotrak 3020, Waterloo, Canada). Each marker consisted of three light-emitting diodes (LED) fixed to a rigid triangle. The triangles were fixed to the participant’s hips and shoulders and to a rigid bar on the platform (Fig. 1a). 3-D LED positions of the triangles were used to calculate marker positions (Fig. 1f, g). Optotrak® and Kistler® output signals as well as the stimulus signals were sampled at 100 Hz using an analogue-digital converter. We recorded all data with software programmed in LabView® (National Instruments, Austin, Texas, USA).

To analyse postural reactions in relation to platform stimuli, transfer functions from stimulus-response data were calculated via a discrete Fourier transform. Fourier coefficients of stimulus and response time series are used to determine *GAIN* and *PHASE* with respect to stimulus frequencies. *GAIN* represents the size of the postural reaction as a function of stimulus size (platform angle), while *PHASE* is related to the relative timing between postural reaction and stimulus [54].

Furthermore, we calculated *COHERENCE*, a measure of reproducibility of the response. Technically, *COHERENCE* is calculated as the quotient between the cross power spectrum of stimulus and response, and the product of the individual spectra of stimulus and response [53]. Whereas a *COHERENCE* value of 0 indicates that there is no linear correlation between the stimulus and response, and 1 indicating a perfect linear correlation with no noise. Values less than 1 occur in practice either because there is noise in the system or there is a nonlinear relation between stimulus and response.

### Parameter identification

Transfer functions served as the experimental data basis for model simulations using a specific version of an established postural control model [36, 49, 53, 55–57] with active time-delayed proportional, derivative, and integral feedback as well as passive stiffness and damping to extract basic constituents of postural control. The physical part of the model is a single inverted pendulum model with corrective torque applied at the ankle joint. The model used here includes a negative feedback loop that relates body excursion detected by visual, vestibular, and proprioceptive sensors to a corrective torque via a neural controller. The neural controller represents the relation between sensory error, ie the difference between the current and desired position on the one hand, and the strength of the motor output, ie torque, on the other hand. With the help of an automated optimization tool (fmincon, MATLAB®, The MathWorks Inc.), which minimized the difference between experimental and simulated GAIN and PHASE curves, we estimated the neural controller’s parameters with proportional (*Kp*), derivative (*Kd*) and integral (*Ki*) contributions (PDI-controller). Neural controller gains are, in part, determined by mass and height of each subject’s center of mass [53]. Because our control group presented lower masses and heights than patients, we had to correct neural controller gains for this effect. That is why we provide numbers for (*Kp/mgh*), (*Kd/mgh*), and (*Ki/mgh*), where mgh represents the gravitational pull (mass)*(gravitational constant)*(height of center of mass). Moreover, we derived time delay (*Td*), proprioceptive sensory weight (*Wp*), and biomechanical elasticity (*Ppas*) and damping (*Dpas*) of the muscles and tendons. We fitted model simulations to experimental transfer functions under different stimulus amplitudes and visual conditions.

### Statistics

Statistical analyses were performed using Microsoft Excel, JMP® and Statview (SAS Institute Inc., Cary, NC, USA). We applied parametric methods after testing the normal distribution and homogeneity of variances with the Kolmogorov-Smirnov test. Due to the expected dependency between experimental conditions and outcome measures, statistical significance was tested by an analysis of variance (ANOVA) for the comparison of healthy subjects and patients. Visual condition, sway direction, and body segment (hip, shoulder) were the within-subjects’ factors for spontaneous sway. For perturbed stance, we applied visual condition, stimulus amplitude, stimulus frequency, and body segment (hip, shoulder) as within-subjects’ factors. For the analysis of the balance based exercise intervention effect on patients, we used a multivariate analysis of variance (MANOVA) with a time as the repeated measure variable, in addition. The level of statistical significance was set at *p* = 0.05.

## Results

No adverse events were observed during the study period. The intervention compliance in terms of number of sessions performed by the patients was 70.1%, mainly due to the underlying disease.

### Spontaneous sway

**Fig. 2:**
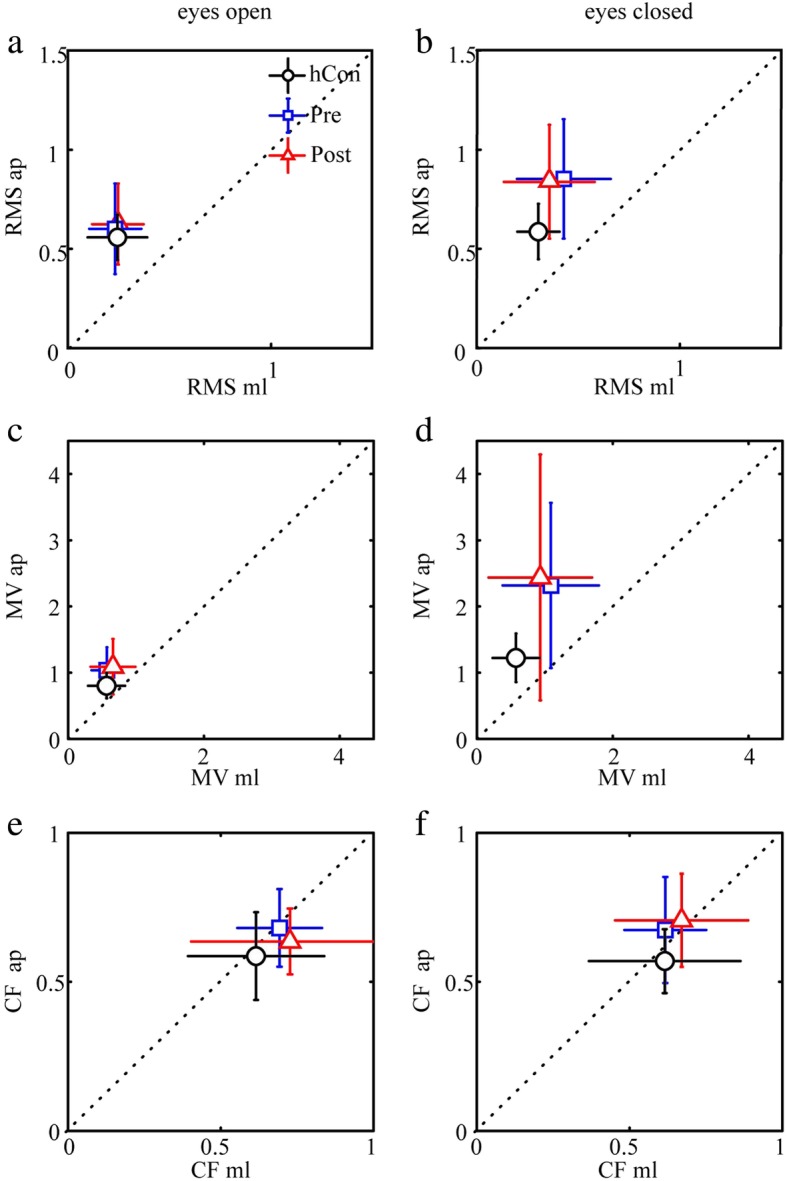
Spontaneous sway. Mean and standard deviation of **a**, **b** root mean square (RMS), **c**, **d** mean velocity (MV) and **e**, **f** center frequency (CF) of COP sway in anterior-posterior (ap) and medio-lateral (ml) direction each for the eyes-open and eyes-closed condition in healthy control subjects (hCon), patients before (pre) and after (post) intervention

The patient group before intervention displayed a significantly larger COP RMS than control subjects (Fig. 2a-b and Table 2). Group designation significantly interacted with sway direction, ie the difference between control subjects and CIPN patients is larger in anterior-posterior direction. Moreover, group designation significantly interacted with visual condition, due to the large RMS in patients with eyes closed. *After intervention*, RMS did not change significantly.

**Table 2:** Spontaneous sway measures (RMS, MV, CF) with group effects, and interactions between groups and visual conditions / sway directions

		Healthy control subjects	Patients pre-intervention	Patients post-intervention	F-value	*p*-value
RMS (cm)		0.46 ± 0.18	0.56 ± 0.27	0.53 ± 0.27	10.926	< 0.0001
visual condition	eyes open eyes closed	0.45 ± 0.18 0.56 ± 0.19	0.49 ± 0.23 0.63 ± 0.30	0.48 ± 0.22 0.59 ± 0.31	5.06	0.007
sway direction	anterior-posterior medio-lateral	0.52 ± 0.16 0.35 ± 0.17	0.67 ± 0.28 0.41 ± 0.19	0.67 ± 0.25 0.35 ± 0.18	4.06	0.018
MV (cm/s)		0.35 ± 0.14	0.69 ± 0.57	0.69 ± 0.56	7.80	0.0005
visual condition	eyes open eyes closed	0.32 ± 0.13 0.39 ± 0.14	0.45 ± 0.31 0.92 ± 0.84	0.50 ± 0.35 0.88 ± 0.81	8.00	0.0004
sway direction	anterior-posterior medio-lateral	0.41 ± 0.13 0.27 ± 0.12	0.82 ± 0.78 0.51 ± 0.44	0.84 ± 0.73 0.49 ± 0.44		n.s.
CF (Hz)		0.37 ± 0.11	0.46 ± 0.18	0.46 ± 0.21		n.s.

*RMS* root means square, *MV* mean velocity, *CF* center frequency, *n.s* not significant

F- and *p*-values refer to the comparison between the three groups and subordinated, to the post-hoc analysis of the interaction with visual condition and sway direction, respectively

As with RMS, the pre-intervention MV of the patient group was significantly larger than in control subjects (Figs. 2c-d, Table 2). The group designation significantly interacted with visual condition (see Table 2): MV values did not differ between groups in the eyes-open condition, whereas the patients’ MV was significantly larger in the eyes-closed condition. *After intervention*, MV did not change.

CF did not differ significantly between patients and control subjects (Figs. 2e-f, Table 2). *After intervention*, patients displayed no effects on CF.

### Perturbed stance

The transfer function between platform tilt and body angular displacement is characterized by GAIN and PHASE behavior.

**Fig. 3:**
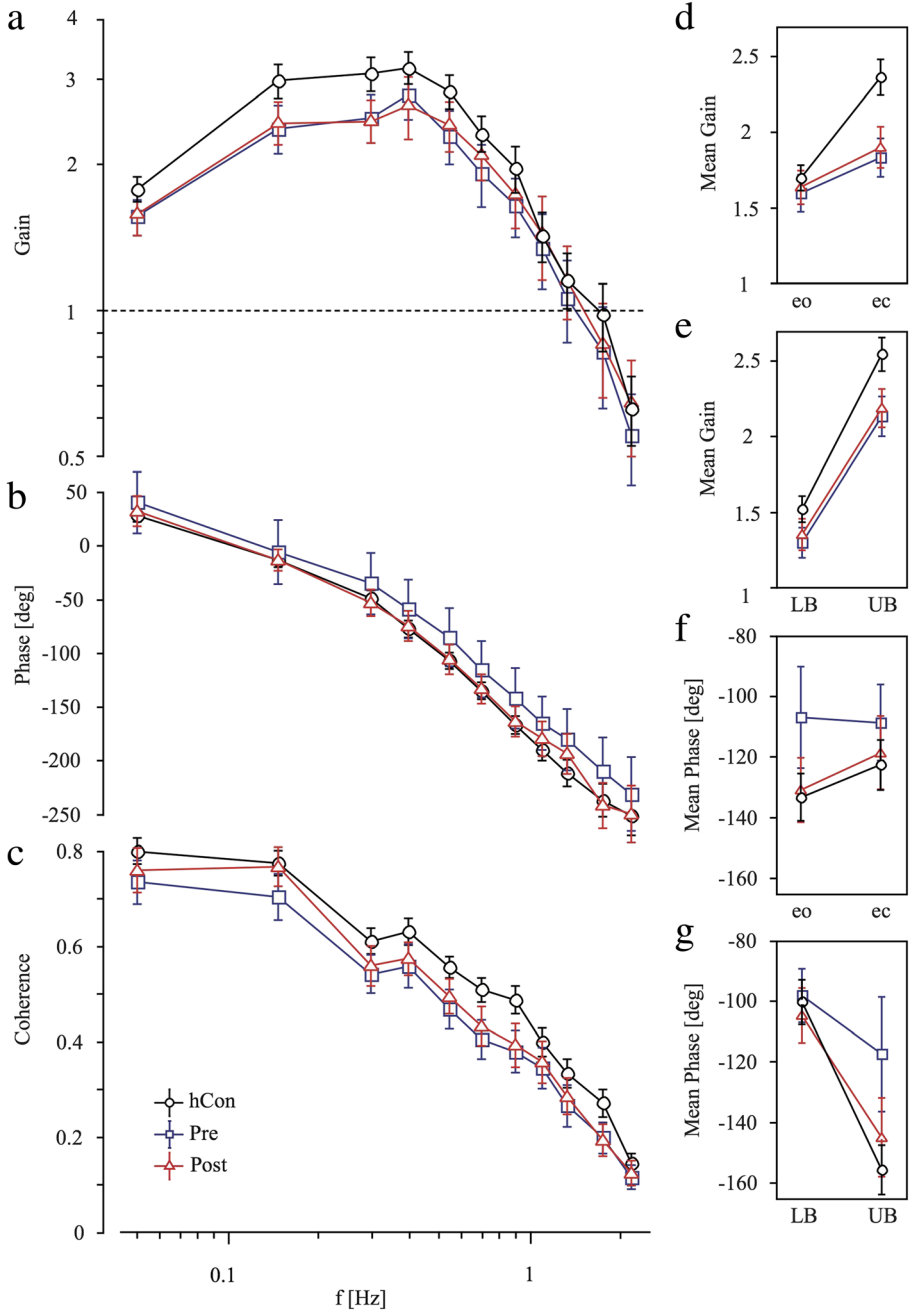
Transfer functions. Mean and standard deviation of **a** Gain, **b** Phase and **c** Coherence behavior as a function of frequency (f) and **d**, **e** mean Gain and **f**, **g** mean Phase behavior for lower body (LB) and upper body (UB) and for eyes-open (eo) and eyes-closed (ec) condition in healthy control subjects (hCon), patients before (pre) and after (post) intervention

**Fig. 4:**
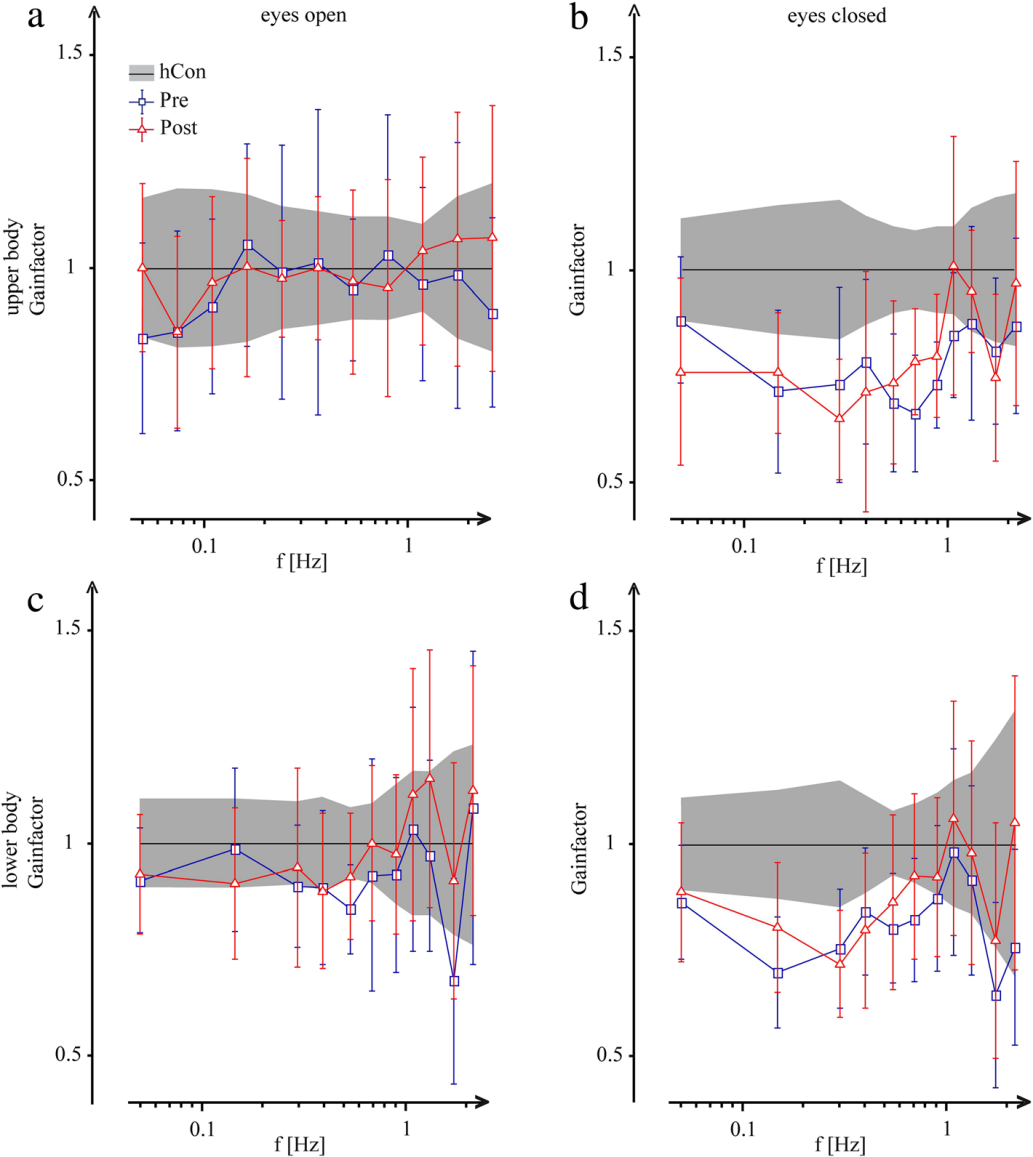
Gainfactor. Mean and standard deviation of **a**, **b** upper body and **c**, **d** lower body GAINFACTOR behavior of patients before (pre) and after (post) intervention as a function of frequency (f). GAINFACTOR represents patients’ GAIN values normalized to values of healthy control subjects (hCon) in the eyes-open and eyes-closed condition

The disturbance-induced body sway, ie GAIN was significantly smaller in patients before intervention (1.57) compared to control subjects (1.87; F = 62.3; *p* < 0.0001; Fig. 3a). *After intervention, patients’ GAIN increased significantly* (1.63; F = 18.0; *p* < 0.0001; Fig. 3a, Fig. 4a-d). Furthermore, group designation interacted significantly with stimulus frequency (F = 3.70; *p* < 0.0001), due to a distortion of the transfer function (Fig. 3a, Fig. 4a-d). Moreover, control subjects’ GAIN is larger with closed eyes than open eyes whereas patients’ GAIN was almost similar independent of the visual condition: group designation significantly interacted with visual condition (eyes open: control subjects 1.58; patients before intervention 1.46; after intervention 1.51; eyes closed: control subjects 2.15; patients before intervention 1.67; after intervention 1.74; visual condition: F = 25.6; *p* < 0.0001, Fig. 3d, Fig. 4a-d). The difference between shoulder and hip sway as a function of platform tilts was greater in control subjects than in patients (Fig. 3e, Fig. 4a-d): with a significant interaction between group designation and body segment (F = 2.85; *p* = 0.022). Group designation and stimulus amplitude did not interact significantly as the effect of stimulus amplitude (non-linearity) on GAIN did not differ between groups.

Concerning PHASE behavior, patients’ PHASE lag before intervention was significantly less pronounced than the control group’s (control subjects -118.3, negative value patients; -107.6, negative value, F = 10.3; *p* < 0.0001; Fig. 3b). *After intervention*, PHASE changed significantly (-121.3, negative value; F = 15.4; *p* < 0.0001; Fig. 3b) and fell in the range of the control subjects’ values (-118.3, negative value; Fig. 3b). Group designation significantly interacted with visual condition (F = 4.45, *p* = 0.01, Fig. 3f): patients with open eyes displayed a PHASE advance of 20 degrees with respect to control subjects, whereas there was no significant PHASE difference between patients and control subjects with eyes closed. Furthermore, group designation significantly interacted with body segment (F = 13.1, *p* < 0.0001, Fig. 3g): the difference between shoulder and hip PHASE was larger in control subjects than in patients. Moreover, group designation significantly interacted with stimulus amplitude (F = 9.89, *p* < 0.0001) as there was a pronounced phase difference with small stimulus amplitudes. Group designation and stimulus frequency did not interact significantly (F = 0.41; *p* = 0.99): the PHASE effects were distributed equally across all frequencies.

COHERENCE as a measure for the reproducibility of the response was smaller in patients before intervention (0.43) compared to control subjects (0.50; F = 103; *p* < 0.0001; Fig. 3c). *After intervention*, COHERENCE did not change (0.45). However, COHERENCE significantly varied with stimulus amplitude, frequency, visual condition, and body segment, similarly in both groups.

### Model-based parameter identification

The following results are derived from the model-based parameter identification procedure [36, 49, 53, 55–57], and present the relevant parameter differences between patients and control subjects.

There was no significant effect for the integral part of the neural controller (Ki), as well as for the proportional (Kp) and derivative part (Kd) of the neural controller.

**Fig. 5:**
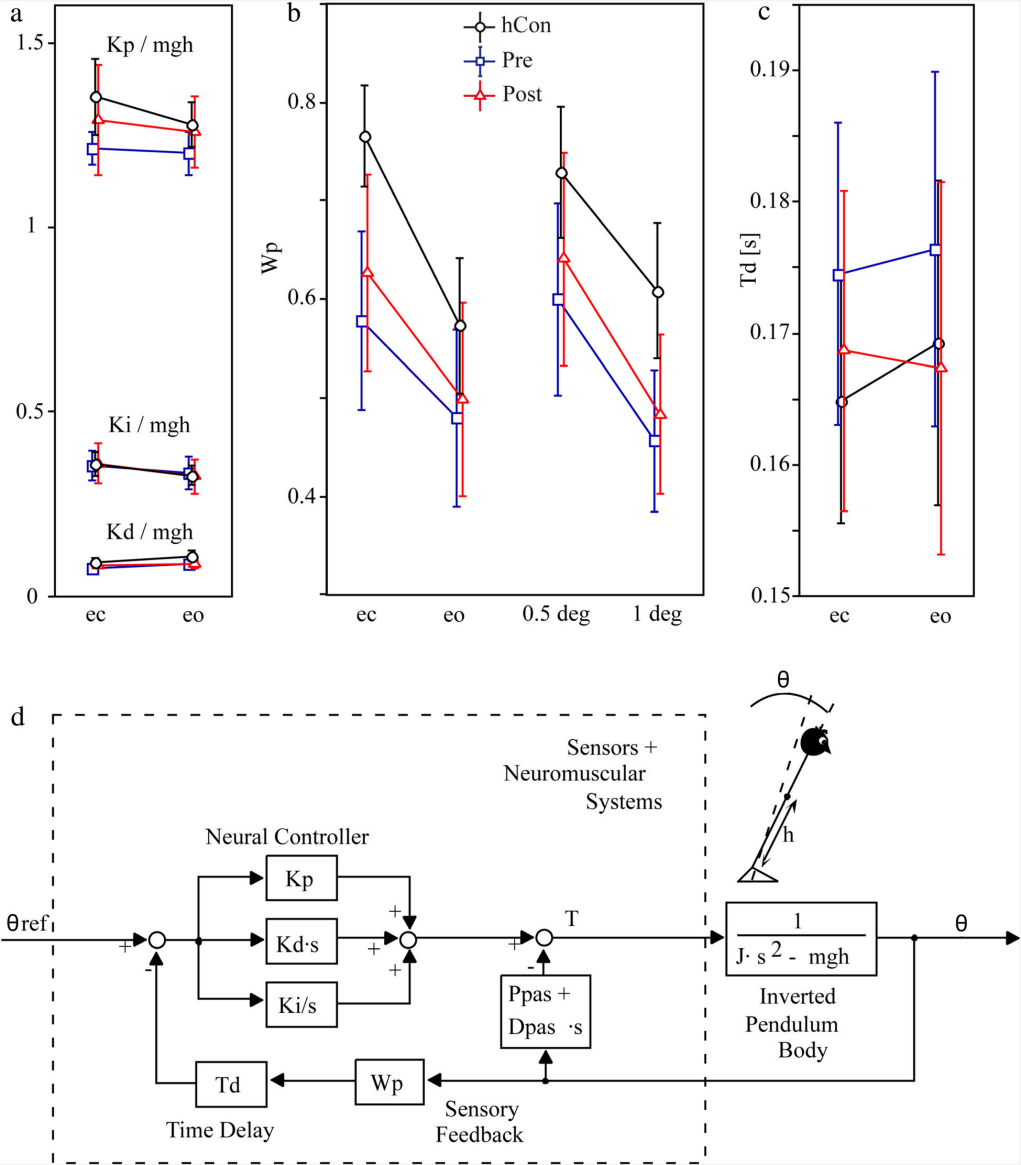
Model parameters. Mean and standard deviation of **a** the neural controller with the proportional (Kp/mgh in 1/°), derivative (Kd/mgh in s/°) and integral (Ki/mgh in 1/s*°) contribution corrected for subjects’ masses and heights, of **b** the proprioceptive sensory weight (Wp in °/°) and **c** the lumped time delay (Td in seconds) for healthy control subjects (hCon), patients before (pre) and after (post) intervention, each shown in the eyes-open (eo) and eyes-closed (ec) condition and for **b** Wp in 0.5 and 1degree (deg) platform rotation. **d** shows the modified postural-control model used to identify abnormal postural control parameters in CIPN patients via an optimization procedure where differences between experimental data and model simulations were minimized: The model consists of a body represented by an inverted pendulum with the mass concentrated at the center of mass of the body and the sensors and neuromuscular systems including a Neural Controller. θ, body sway angle; h, height of the center of mass above the ankle joints; θ ref., external stimulus; Kp, proportional gain (stiffness factor), Kd, derivative gain (damping factor), Ki, integral gain of the Neural Controller; Ppas, passive stiffness factor; Dpas, passive damping factor; Wp, proprioceptive sensory weight; Td, feedback time delay; T, control torque; J, moment of inertia of the body; mgh, body mass*gravitational constant*height of the center of mass from the ankle joint; s, Laplace transform variable

The sensory weighting factor Wp differed significantly between patients and control subjects (F = 9.89, *p* = 0.0001; Fig. 5b). Whereas patients rely with an average factor of 0.53 on proprioceptive cues and hence 0.47 on spatial cues, control subjects rely with a factor of 0.67 on proprioceptive and 0.33 on spatial cues. Group designation did not interact with visual condition or stimulus amplitude. *After intervention*, there was a small but significant change towards control subjects (Wp 0.56, F = 9.13, *p* = 0.006).

The time delay between stimulus and response (Td) did not differ significantly between patients and control subjects (F = 1.10; *p* = 0.34; Fig. 5c). *After intervention*, Td was not affected.

Parameters related to passive muscle and tendon behavior (Ppas and Dpas) did not differ significantly between groups. *After intervention,* these parameters were not affected.

Figure 5d shows the modified postural-control model.

## Discussion

As postural instability is a momentous symptom of CIPN [9–16], the first aim of this study was to assess the specific set of postural control deficits associated with CIPN compared to healthy subjects. Furthermore, since CIPN treatment options are very limited so far [3] and hints in the recent literature indicate that CIPN patients might benefit from exercising [39, 41, 58], we evaluated a balance-based exercise intervention aiming to treat patients’ functional impairments due to CIPN. While former studies mostly investigated spontaneous sway measures (displacement-, velocity-, and frequency-related measures), we aimed to describe CIPN patients’ sensorimotor behavior in much greater detail. Therefore, we additionally analyzed patients’ stance behavior as reaction to an external perturbation (following a pseudorandom stimuli) by generating transfer functions between body behavior and stimuli.

### Spontaneous sway

Concerning spontaneous sway, we found greater postural sway in CIPN patients similarly to previous CIPN studies [10, 12, 14, 39]. Additionally, our findings correspond to that of other types of neuropathy. For example, many working groups [59–63] report increased RMS and MV in patients with diabetic-induced neuropathy. In our study, RMS and MV were significantly larger in CIPN patients than in healthy subjects. Moreover, we observed a specific preponderance of deficits in anterior-posterior direction [60] and a more pronounced postural sway with closed rather than open eyes [60, 61]. Generally, closing their eyes causes subjects to use vestibular and proprioceptive cues for controlling balance. As proprioceptive information are often deficient in neuropathy patients [26], it seems reasonable to assume that patients may prefer vestibular over proprioceptive cues. However, it is well known that the vestibular signal carries a larger amount of noise than the proprioceptive signal [64] leading to less accurate posture control. We speculate at this point that the main source for the larger RMS and MV is related to a sensory shift towards vestibular cues (see below sensory weighting, and [65]). If that is true, we might be able to recover this finding when dissociating proprioceptive from vestibular frames of orientation using platform tilts.

### Perturbed stance

Whereas proprioceptive cues may drag the body along platform movements, quantified by a relative larger GAIN as transfer function between body excursions and platform tilts, vestibular cues would stabilize the body in space, quantified by a relative smaller GAIN. In fact, CIPN patients presented smaller GAIN values since their reaction to platform tilts were less pronounced than that of control subjects. Thus, they might rather use space coordinates than platform movements for posture control. Furthermore, GAIN was significantly affected by visual condition and body segment. The larger GAIN difference between CIPN patients and control subjects in the eyes-closed compared to the eyes-open condition suggests that under-usage of proprioception is dominant when there are less additional orienting cues. Moreover, the finding of relatively small GAINs in CIPN patients’ lower compared to their upper body segment point to a slightly different intersegmental strategy [29]. Our PHASE finding, that the difference between shoulder and hip PHASE was larger in control subjects than in patients, also points to a different intersegmental behavior in terms of upper with respect to lower body angular displacements [26, 28, 29]. We assume that patients proactively orientate themselves, especially their upper body, more towards space coordinates. This also indicates an especially low use of proprioception according to our GAIN results. Moreover, we speculate that CIPN patients proactively assume a safety strategy that may follow an enhanced muscle co-contraction [25], leading to smaller body excursions. However, greater co-contraction limits one’s ability to precisely control posture [66, 67].

### Model-based parameter identification

To address the transfer-function abnormalities in CIPN patients, we fitted the subjects’ data via a simple feedback system [36, 56, 64, 68]. Using the model-based parameter estimation, we identified and quantified the CIPN patients’ diminished use of proprioceptive cues: The sensory-weighting factor for proprioception (Wp) is significantly smaller in CIPN patients than in control subjects. However, patients did not present a different error correction gain (Kp and Kd) of the feedback loop. Furthermore, parameters related to passive muscle and tendon behavior (passive stiffness and damping, Kpas and Bpas) did also not differ between CIPN patients and control subjects. This seems to be in line with the notion that differences in postural control between CIPN patients and healthy subjects mainly rely on active postural control differences related to the different use of sensory cues, whereas passive muscle and tendon characteristics are not significantly affected by CIPN. Moreover, this finding might indicate that muscles and tendons were not affected supporting CIPN’s primarily sensory characteristic.

### Intervention effects

Our patients performed more than two third of the prescribed exercise sessions. Considering the high variety of our patient group with different diagnosis and disease severities, we assume a reasonable coherence rate that lies in the range of other interventional studies with cancer patients in general [69] or CIPN patients specifically [41].

How does the exercise intervention influence CIPN patients’ postural behavior? Interestingly, we observed that CIPN patients’ main abnormality (down-weighted proprioception) was modified by the exercise intervention. CIPN patients’ GAIN and PHASE values reached those of healthy subjects due to the proprioceptive up-weighting mentioned above. Interestingly, the effect of up-weighting proprioception is the only significant model-based parameter modification after intervention. Why would CIPN patients profit from up-weighting proprioception while suffering from a supposed proprioceptive deficit? Our clinical assessments did not suffice to conclusively specify neural lesions or identify CIPN’s nerve fiber contribution occurring in our patients. However, all patients suffered from strong paresthesia and reported significant balance problems confirmed by our spontaneous sway experiments. CIPN sensory symptoms are described to refer to ‘terminal arbor degeneration’ and the preferential damage of myelinated primary afferent sensory nerve fibers [2, 70]. Therefore, we speculate that patients’ peripheral information and subsequent stimulus conduction are altered, but not completely dysfunctional. The down-weighting of proprioceptive cues could be interpreted as an excessive compensatory mechanism, which lets CIPN patients pre-intervention remain in a suboptimal state. The exercise intervention may thus partially correct this excess and may stimulate the use of less damaged pathways. As a side effect of proprioceptive up-weighting, the intervention may trigger down-weighting of vestibular cues, thereby reducing vestibular noise. As a consequence, postural-control behavior might be more accurate in terms of less variability.

Conclusively, we maintain that up-weighting proprioception and thereby down-weighting vestibular information towards the behavior of healthy subjects represents a clear benefit for CIPN patients. The proprioceptive cue is considered to be more accurate than vestibular cues for postural stability [64]. Regarding time delay, patients after intervention tended to improve their reaction time between stimulus and response. We also identified a tendency of spontaneous sway RMS values to be smaller after intervention, being in line with postural sway findings after balance training in a study of Schwenk et al. [39]. Although, our intervention effects are small, we are convinced that CIPN patients benefit from exercising in terms of improved posture behavior that substantially contributes to patients’ functional status. Functional performance is an acknowledged prognosis factor for cancer survivor [71] why we strongly propose to verify our intervention results in a greater randomized controlled trial. Our findings are limited by the small sample size and the lack of patient control group. Furthermore, expanding neurophysiological assessments would provide insights in adaptive processes unexamined in this study.

## Conclusion

We believe that our new approach contributed to a deeper understanding of CIPN patients’ postural instability. Proprioceptive down-weighting might represent the main postural deficit in CIPN. Our exercise intervention targeted specifically this abnormality presumably by primarily correcting the overactive compensation, which led to a significant improvement in postural stability. We believe that a balance-based exercise intervention is a promising strategy to manage functional impairments due to CIPN and that it should therefore be routinely integrated within the treatment regimens of patients receiving neurotoxic agents.

## Data Availability

The dataset supporting the conclusions of this article is included within this article. The data that support the findings of this study are available from the corresponding author upon reasonable request.

## Abbreviations

CF: Center frequency
CIPN: Chemotherapy-induced peripheral neuropathy
COP: Center of pressure
Dpas: Passive damping
Kd: Derivative contribution of the neural controller
Ki: Integral contribution of the neural controller
Kp: Proportional contribution of the neural controller
MV: Mean velocity
NtxS: Neurotoxicity subscale of FACT&GOG
Ppas: Passive stiffness
RMS: Root means square
Td: Time delay
Wp: Proprioceptive sensory weight
